# Two different pathogenic gene mutations coexisted in the same hereditary spherocytosis family manifested with heterogeneous phenotypes

**DOI:** 10.1186/s12881-019-0826-7

**Published:** 2019-05-24

**Authors:** Hongwei Shen, Hui Huang, Kaizhong Luo, Yan Yi, Xiaoliu Shi

**Affiliations:** 1 Central Lab, The Second Xiangya Hospital, Central South University, Changsha, Hunan Province People’s Republic of China; 2 Department of Medical Genetics, The Second Xiangya Hospital, Central South University, Changsha, Hunan Province People’s Republic of China; 3 Department of Infectious Diseases, The Second Xiangya Hospital, Central South University, Changsha, Hunan Province People’s Republic of China; 4 Department of Hematology, The Second Xiangya Hospital, Central South University, 139 Middle Renmin Road, Changsha, Hunan Province 410011 People’s Republic of China; 5 Department of Medical Genetics, The Second Xiangya Hospital, Central South University, 139 Middle Renmin Road, Changsha, Hunan Province 410011 People’s Republic of China

**Keywords:** Hereditary spherocytosis, Genetic diagnosis, Whole-exome sequencing, Heterogeneous genotype, Heterogeneous phenotype

## Abstract

**Background:**

Hereditary spherocytosis (HS) is a common type of hereditary hemolytic anemia. According to the current diagnostic criteria of HS, patients with a family history of HS, typical clinical features and laboratory investigations could be diagnosed without the requirement of any additional tests, including genetic analysis. However, the clinical heterogeneities incur difficulties in HS diagnosis. We therefore aimed to investigate the application of genetic diagnosis in a family-based cohort.

**Case presentation:**

In the present Chinese family, two probands sharing similar clinical manifestations, including jaundice, cholelithiasis, splenomegaly and spherocytes, while the clinical features of other family members were inconclusive. Whole-exome sequencing (WES) unexpectedly unveiled two separate disease-causing mutations in the two probands. *SPTB* R1625X mutation detected in proband D was a *de novo* mutation; while proband W inherited the *SLC4A1* c.G1469A mutation from her mother, which was also inherited by her brother. However, the clinical features of proband W and her mother and brother were discrepant: proband W suffered from significant splenomegaly, jaundice and cholelithiasis, which resulted in cholecystectomy and splenectomy; while her mother and brother’s HS were not complicated by cholelithiasis, and their splenomegaly and elevated serum bilirubin were moderate. In addition, additional genomic defects involved with HS-related symptoms have not been detected in this family.

**Conclusions:**

Both genotypes and phenotypes could be heterogeneous in the same HS family. The analysis of pathogenic gene mutations may endeavor to play an indispensable role in the accurate diagnosis and genetic consultation of HS individuals and their family members.

## Background

Hereditary spherocytosis (HS) is a common type of hereditary hemolytic anemia in all racial and ethnic groups and is characterized by the presence of spherical-shaped erythrocytes (spherocytes) on the peripheral blood smear [1]. The clinical manifestations of typical HS include hemolytic anemia, jaundice, splenomegaly and cholelithiasis [1]. According to the diagnostic criteria of HS, patients with a family history of HS, typical clinical features and laboratory investigations (spherocytes, raised mean corpuscular hemoglobin concentration [MCHC], reticulocytosis) could be diagnosed without the requirement of any additional tests [2]. However, the HS clinical spectrum ranges widely from an almost silent phenotype to severe life-threatening anemia, significant splenomegaly, or extreme hyperbilirubinemia, even in the same family [1, 2]. The clinical heterogeneities incur difficulties in HS diagnosis. Additionally, definitive diagnosis may be difficult when testing results are not consistent or conclusive [3, 4].

HS is usually inherited in an autosomal dominant manner. Pathogenic mutations involve the *ankyrin 1* (*ANK1*) gene on chromosome 8p11, the *spectrin β* (*SPTB*) gene on chromosome 14q23, the *spectrin α erythrocytic 1* (*SPTA1*) gene on chromosome 1q21, the *solute carrier family 4 member 1* (*SLC4A1*) gene on chromosome 17q21, and the *erythrocyte membrane protein band 4.2* (*EPB42*) gene on chromosome 15q15. The advent of whole-exome sequencing (WES) has made molecular diagnosis of hereditary erythrocyte membrane defects, including HS, feasible [5, 6]. However, molecular analysis of the affected genes has not been recommended as one of the routine diagnostic approaches by current HS guidelines [2].

Here, we present an HS family in which two disease-causing mutations, *SPTB* c.C4873T and *SLC4A1* c.G1469A, were identified separately in different individuals. In this family, there was no correlation between genotype and phenotype: the individuals harboring the same pathogenic gene mutation presented with different manifestations, while the individuals who presented with similar clinical features harbored different pathogenic mutations. The analysis of pathogenic gene mutations played an indispensable role in the accurate diagnosis and genetic counseling of this family.

## Case presentation

Proband D, a 26-year-old man, complained of recurrent jaundice for 8 years and splenomegaly for more than 6 years. Physical examination revealed cutaneous and icteric sclera; the spleen was palpable 60 mm below the costal margin. His serum total bilirubin (TBIL) was 73.1 μmol/l and his direct bilirubin (DBIL) was 7.3 μmol/l. The complete blood count revealed hemoglobin 125 g/l, reticulocytes 0.334 × 10^12^/l, mean corpuscular volume (MCV) 85.7 fl, mean corpuscular hemoglobin (MCH) 28.4 pg and MCHC 332 g/l, and spherocytes accounted for 13.6% of red blood cells (RBCs). Abdominal ultrasonography detected cholelithiasis in addition to splenomegaly. Serum hepatitis B virus surface antigen was positive, while liver biopsy showed no cirrhosis (Table 1).

**Table 1:** Clinical and genetic features of proband D, proband W and their immediate family members

	proband D before splenectomy	proband D after splenectomy	proband D’s father	proband D’s mother	proband W before splenectomy	proband W after splenectomy	proband W’s father	proband W’s mother	proband W’s brother
Age (Y)	25	26	50	49	20	24	48	48	18
Sex	M	M	M	F	F	F	M	F	M
Spleen (mm below rib)	60	/	Not palpable	Not palpable	100	/	Not palpable	32	27
Cholelithiasis	Y	/	N	N	Y	/	N	N	N
HBV carrier	Y	Y	Y	N	N	N	N	NA	N
HB (g/l)	125	181	152	125	114	163	159	114	150
MCV (fl)	85.7	83.7	88.7	91.4	79.8	88.4	91.2	84.7	92.5
MCH (pg)	28.4	27.1	29.2	29.9	29.9	32.0	31.2	30.1	33.0
MCHC (g/l)	332	323	329	327	374	364	343	355	356
Ret (×10^12^/l)	0.334	NA	0.084	0.061	0.373	0.081	0.138	0.145	0.188
Spherocyte (%)	13.6	NA	8	1	15	30	10	19.6	18
TBIL (μmol/l)	73.1	19.1	12.8	7.6	74.0	27.3	19.3	31.7	29.2
DBIL (μmol/l)	7.3	8.3	4.2	2.9	19.4	7.3	5.7	12.6	12.1
LDH (U/l)	224.9	NA	239	171.9	226.4	869.9	254.4	188.7	307.4
*SPTB* c.4873 C > T p.R1625X		Y	N	N		N	N	N	N
*SLC4A1* c.1469G > A p. R490H		N	N	N		Y	N	Y	Y

*HB* hemoglobin, *MCV* mean corpuscular volume, *MCH* mean corpuscular hemoglobin, *MCHC* mean corpuscular hemoglobin concentration, *Ret* reticulocytes, *TBIL* total bilirubin, *DBIL* direct bilirubin, *LDH* lactate dehydrogenase, *SPTB spectrin β*, *SLC4A1 solute carrier family 4 member 1*, *M* male, *F* female, *Y* Yes, *N* No, *NA* not available

Proband W, a 24-year-old girl, was diagnosed with HS complicated with jaundice and cholelithiasis and underwent cholecystectomy and splenectomy less than 5 years ago. Before the operation, her spleen was palpable 100 mm below the costal margin. Her serum TBIL was 74.0 μmol/l, and her DBIL was 19.4 μmol/l. The complete blood count revealed hemoglobin 114 g/l, reticulocytes 0.373 × 10^12^/l, MCV 79.8 fl, MCH 29.9 pg and MCHC 374 g/l. Spherocytes accounted for 15.0% of RBCs (Table 1). She was re-evaluated clinically. Her TBIL was 27.3 μmol/l, and her DBIL was 7.3 μmol/l. Her hemoglobin was 163 g/l, reticulocytes was 0.081 × 10^12^/l, MCV was 88.4 fl, MCH was 32.0 pg, and MCHC was 364 g/l. Spherocytes accounted for 30.0% of RBCs (Table 1).

Proband D and proband W are cousins (their mothers are sisters). Of the proband W’s immediate family members, splenomegaly and elevated reticulocytes, spherocytes and serum bilirubin were detected in her mother and brother. Her mother’s spleen was palpable 32 mm below the costal margin. Her TBIL was 31.7 μmol/l, and her DBIL was 12.6 μmol/l. her was hemoglobin 114 g/l, reticulocytes was 0.145 × 10^12^/l, MCV was 84.7 fl, MCH was 30.1 pg and MCHC was 355 g/l. Spherocytes accounted for 19.6% of RBCs. Her brother’s spleen was palpable 27 mm below the costal margin. His TBIL was 29.2 μmol/l, and his DBIL was 12.1 μmol/l. his hemoglobin was 150 g/l, reticulocytes was 0.188 × 10^12^/l, MCV was 92.5 fl, MCH was 33.0 pg, and MCHC was 356 g/l. Spherocytes accounted for 18.0% of RBCs. In addition, slightly elevated reticulocytes (0.138 × 10^12^/l), serum bilirubin (TBIL19.3 μmol/l) and spherocytes (10.0% of RBCs) also existed in proband W’s father, while his spleen was not enlarged (details in Table 1).

However, no splenomegaly was found in proband D’s immediate family members. Only slightly elevated reticulocytes (0.084 × 10^12^/l) and spherocytes (8.0% of RBCs) were detected in his father. No HS clinical features or laboratory findings, such as spherocytes, raised MCHC or reticulocytosis, were found in his mother, who is the sister of proband W’s mother (details in Table 1).

Proband D’s father was also discovered to be a hepatitis B virus (HBV) carrier. Elevated serum lactate dehydrogenase (LDH) (307.4 U/L) was detected only in proband W’s brother but not in any other family members (Table 1).

Peripheral vein blood was drawn from the probands and their immediate family members after they signed informed consents. The genomic DNA was extracted and the genomic DNA library was constructed according to Agilent’s protocol. WES services were provided by the Chigene Bioinformatics Institute (Beijing, China). Exomes were captured by Nimblegen kits, and next-generation sequencing (NES) was conducted with an Illumina HiSeq. The results were aligned to the University of California Santa Cruz, human genome assembly 19 (UCSC.hg19; http://genome.ucsc.edu/) reference sequence. VCFtools program of the SAMTools software, version 0.1.16 (http://samtools.sourceforge.net/) was used to identify and call variants, including single-nucleotide polymorphisms (SNPs) and indels. ANNOVAR was used to annotate the variants. There are 48,747 variants and 49,276 variants were identified in proband D and proband W respectively. The strategies of data filtering were on the basis of the published documents [7, 8].

**Fig. 1:**
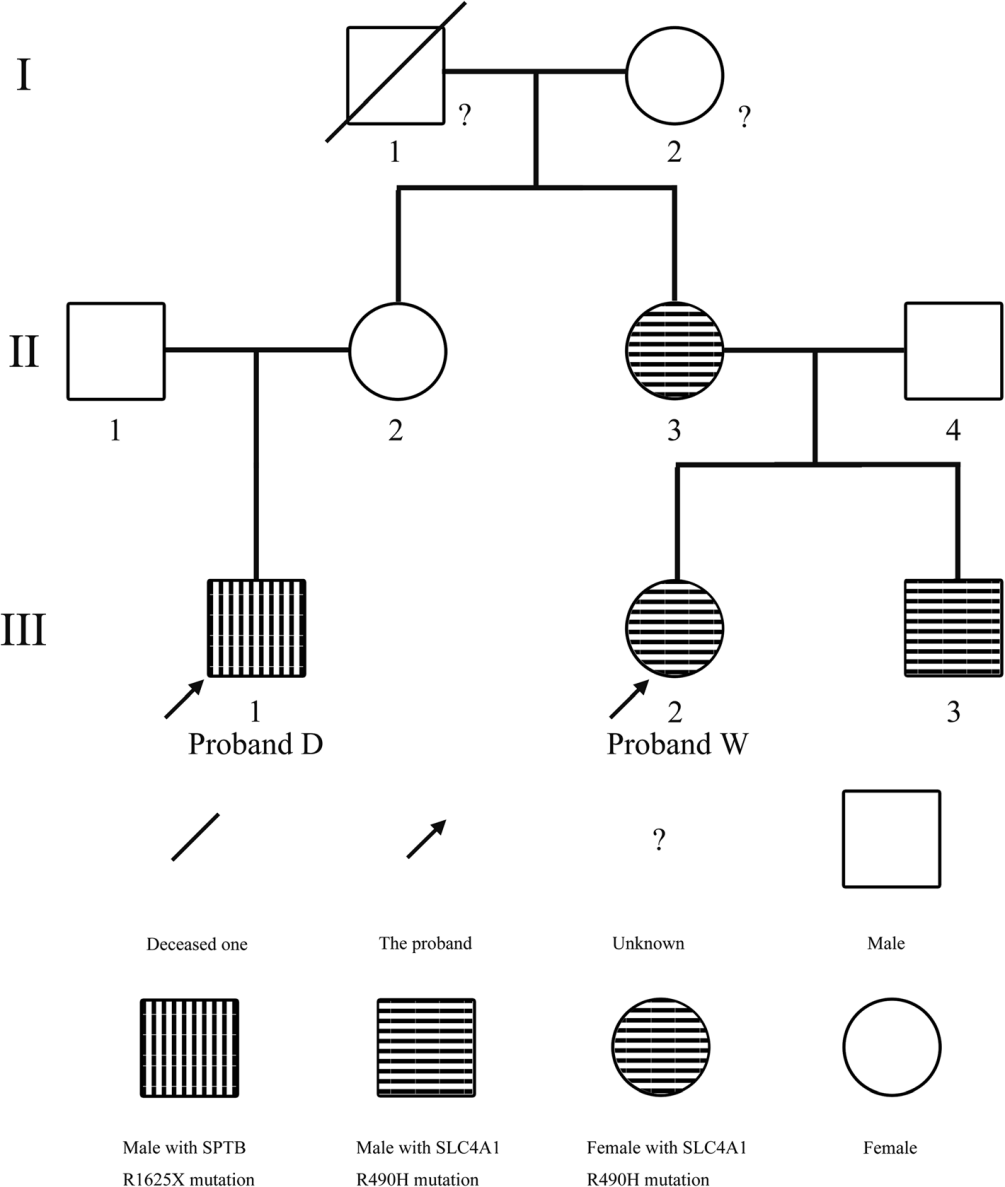
Pedigree of the present family with hereditary spherocytosis (HS)

**Fig. 2:**
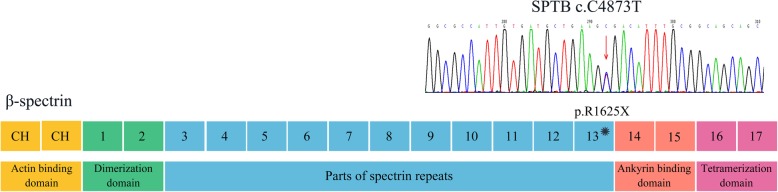
Sanger sequencing of the pathogenic *SPTB* c.C4873T mutation in proband D and the localization of the mutation in schematic diagram of β-spectrin. A heterozygous c.C4873T (p.R1625X) mutation in *SPTB* gene, which encodes β-spectrin, was identified in proband D. Human erythroid β-spectrin molecule consists of two N-terminal calponin homology (CH) domains responsible for actin binding and seventeen β-spectrin repeats including dimerization domain (repeats 1 and 2), Ankyrin binding domain (repeats 14 and 15) and a tetramerization domain (repeats 16 and 17). The nonsense R1625X mutation was located on β-spectrin repeat 13. The red arrow indicates C4873T mutation detected in Sanger sequencing. The asterisk indicates the position of R1625X mutation in β-spectrin. *SPTB*, *spectrin β*

WES revealed that proband D was heterozygous for the c.C4873T (p.R1625X) mutation within exon 23 of *SPTB*, which were confirmed by Sanger sequencing of the polymerase chain reaction (PCR) products. Primers are as following: forward 5′- GGTCTCTCAAAGCTGGAATGA − 3′, reverse 5′- AAATTGGTGCTCTCAGGTCA -3′. PCR products were sequenced and analyzed using Phred-Phrap-Consed version 12.0 software (http://phrap.org/phredphrapconsed.html). Sequencing results were compared with the reference sequences for *SPTB* (GenBank accession no. NM_001024858). Then, the detected mutations were searched in the 1000 Genomes Project database and Exome Aggregation (http://internationalgenome.org/1000-genomes-browsers). The *SPTB* R1625X mutation was not present in any of his immediate family members (Fig. 1). This mutation introduced a premature stop codon at amino acid residue 1625, which created a truncated β-spectrin protein, and was predicted to be disease-causing by the analytical software. The truncated protein lack of the ankyrin binding domain and the tetramerization domain has been proved to lead to inefficient incorporation of the mutant protein into the skeleton and result in HS (Fig. 2) [9–11]. In addition, it has been previously identified as a pathogenic mutation [12, 13]. Therefore, combined with the patient’s clinical diagnosis of HS, the *SPTB* R1625X was identified as a pathogenic mutation, and was causative for HS development in proband D according to the American College of Medical Genetics (ACMG) guidelines [14]. The *SPTB* R1625X mutation was determined to be a *de novo* mutation in proband D.

**Fig. 3:**
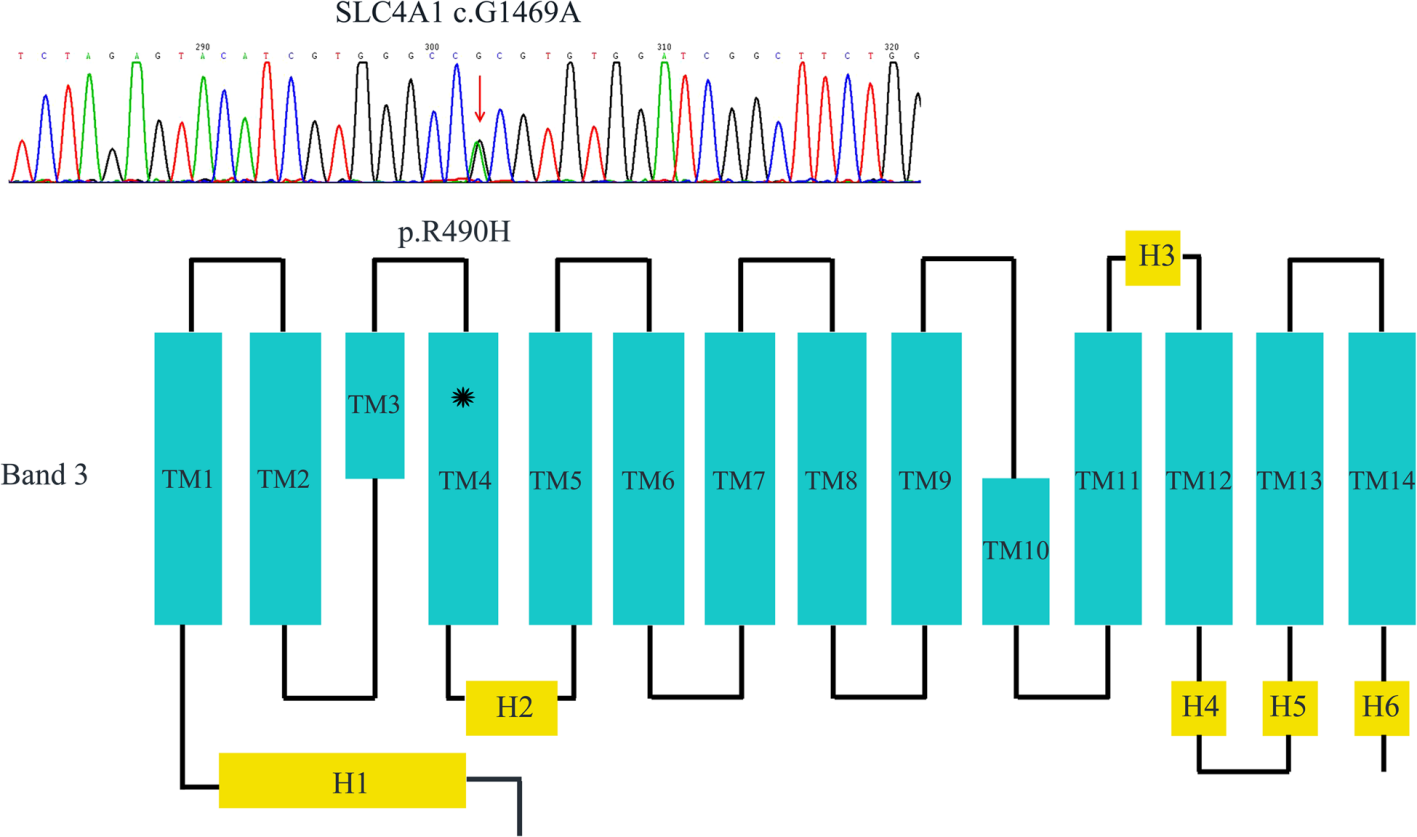
S Sanger sequencing of the pathogenic *SLC4A1* c.G1469A mutation in proband W and the localization of the mutation in schematic diagram of band 3. A heterozygous c.G1469A (p.R490H) mutation in *SLC4A1* gene, which encodes band 3, was identified in proband W. Band 3 consists of 14 transmembrane (TM) segments and short helical (H) segments linking TM segments. The missense R490H mutation was located on TM4. The red arrow indicates G1469A mutation detected in Sanger sequencing. The asterisk indicates the position of R490H mutation in band 3. *SLC4A1*, *solute carrier family 4, anion exchanger, member 1*

Meanwhile, WES revealed that proband W was heterozygous for the c.G1469A (p.R490H) mutation within exon 13 of *SLC4A1*, which was confirmed by Sanger sequencing of the PCR products. Primers for PCR were as following: forward 5′-TTGACTGACCGGCTTCTTCT-3′, reverse 5′-AGGACACAATGGCTCAGTCT-3′. Sequencing results were compared with the reference sequences for *SLC4A1* (GenBank accession no. NM_000342). The *SLC4A1* R490H was also present in her mother and brother (Fig. 1). *SLC4A1* gene codes band 3 protein, the predominant glycoprotein of RBCs membrane. Band 3 consists of 14 transmembrane (TM) segments and short helical (H) segments linking TM segments. HS-related mutations are common in the TM segments. This mutation predicted a change from arginine to histidine at highly conserved amino acid residue 490, which was located at the N-terminus boundary of the fourth putative TM segment of band 3, and the substitution could destabilize the helix and position of the segment in the bilayer of red blood cell membrane (Fig. 3) [15]. And the mutation was predicted to be disease-causing by the analytical software. In addition, it has been previously reported as a pathogenic mutation [16, 17]. Therefore, combined with the patient’s clinical diagnosis of HS, *SLC4A1* R490H was probably as a pathogenic mutation and was causative for HS development in proband W, her mother and brother according to the ACMG guidelines [14].

In addition, with the combination of WES and Sanger sequencing, no additional genomic defects involved in HS-related symptoms, such as uridine diphosphate glucuronosyl transferase 1A1 (UGT1A1) deficiency involved in jaundice, have been detected in the whole family.

## Discussion and conclusions

HS is a common hereditary hemolytic anemia with a heterogeneous phenotype that arises from reduced deformability due to defects in several cytoskeletal proteins, including ankyrin, α spectrin, β spectrin, band 3 and protein 4.2 [1]. As HS pathogenic mutations involve five large-size genes, labor-intensive Sanger sequencing has been precluded for HS molecular diagnosis. In recent years, WES has shed new light on HS diagnosis and evaluation as a comprehensive and rapid diagnostic tool, especially in cases where traditional testing has failed, or was inconclusive, although WES has not been accepted as a routine diagnostic tool [6, 7, 12, 18].

In the present study, proband D and proband W presented with similar manifestations including jaundice, cholelithiasis and splenomegaly. Both probands underwent splenectomy and cholecystectomy. Since their mothers were sisters, their HS had been predicted to be inherited from their maternal side. In further clinical analysis, moderate HS manifestations were detected in proband W’s mother and brother. However, no HS manifestations were found in proband D’s mother, while the inconclusive values near cut-off points including reticulocyte, serum bilirubin and spherocytes, were also detected in healthy family members, proband W and proband D’s fathers. Hence, according to the clinical data, the identification of HS individuals and the pattern of inheritance in this family was obscure.

To clarify the HS diagnosis and genetic model, WES was performed on samples from this family. Surprisingly, two different HS pathogenic mutations, *SPTB* c.C4873T and *SLC4A1* c.G1469A, were identified in proband D and proband W separately. Furthermore, *SPTB* c.C4873T was confirmed as a *de novo* mutation in proband D, while in proband W, HS was attributed to *SLC4A1* c.G1469A and determined to be inherited from her mother and was also inherited by her brother. Other relatives were excluded as HS patients. Sanger sequencing confirmed the findings of WES. Notably, although proband W, her mother and brother shared the same pathogenic mutation, *SLC4A1* c.G1469A, their clinical features were discrepant: proband W suffered from significant splenomegaly, jaundice and cholelithiasis, which incurred cholecystectomy and splenectomy; in her mother and brother, their HS was not complicated by cholelithiasis, and their splenomegaly and elevated serum bilirubin were moderate. Additional genomic defects involved in HS-related symptoms have not been detected in this family. And the reason of their clinical discrepancy is unknown.

In this family, analysis of clinical manifestations was not conclusive for the identification of HS individuals and the pattern of inheritance, while genetic analysis combined WES and Sanger sequencing provided accurate evidence for precise diagnosis, and clarified the complex genetic background of this family. Although current guidelines do not recommend genetic analysis for routine HS diagnosis, especially for newly diagnosed patients with a family history of HS [1], in our experience, HS presents with wide heterogeneity in clinical symptoms, and genetic analysis is a time- and cost-effective method that gave a clear conclusion to the obscured individuals without typical clinical and laboratory features in this HS family. In particular, genetic analysis was indispensable for the identification of the pathogenic mutation and pattern of inheritance and could sometimes unveil unexpected pathogenic evidence, which is also very meaningful for investigating the precise underlying pathophysiologic mechanism of HS individuals or families.

In addition, according to published mutations of pathogenic HS genes, there are no hotspot mutations, and most mutations are sporadic [19]. Studies on genotype-phenotype relationships of HS have been attempted [10, 16]. However, no definite conclusions have been drawn. Moreover, because HS is an autosomal dominant genetic disease, de novo mutations should not be rare in HS. Hence, analysis of the pathogenic mutations of HS is complex. WES is a comprehensive and invaluable diagnostic tool that overcomes the limitations of the detection capability of Sanger sequencing, making the thorough and individualized evaluations of specific HS individuals feasible. In addition, during interpretation on the pathogenicity of the mutations detected by WES, ACMG guidelines should be followed rigorously to avoid incorrect interpretation of HS-related gene mutations, and provide precise diagnosis and genetic consultation for HS patients and their family members.

In conclusion, *de novo* *SPTB* c.C4873T mutation and inherited the *SLC4A1* c.G1469A were identified separately, in the present HS family. Moreover, the individuals harboring the same pathogenic gene mutation presented with different manifestations, while the individuals who presented with similar clinical features harbored different pathogenic mutations. Our findings suggested that both genotypes and phenotypes could be heterogeneous in the same HS family. The analysis of pathogenic gene mutations combined WES and Sanger sequencing is a time- and cost-effective method that may endeavor to play an indispensable role in the accurate diagnosis and genetic consultation of HS individuals and their family members.

## Abbreviations

ACMG: American College of Medical Genetics
ANK1: Ankyrin 1
DBIL: Direct bilirubin
EPB42: Erythrocyte membrane protein band 4.2
H: Helical
HBV: Hepatitis B virus
HS: Hereditary spherocytosis
LDH: Lactate dehydrogenase
MCH: Mean corpuscular hemoglobin
MCHC: Mean corpuscular hemoglobin concentration
MCV: Mean corpuscular volume
PCR: Polymerase chain reaction
RBCs: Red blood cells
SLC4A1: Solute carrier family 4 member 1
SNPs: Single-nucleotide polymorphisms
SPTA1: Spectrin α erythrocytic 1
SPTB: Spectrin β
TBIL: Total bilirubin
TM: Transmembrane
UGT1A1: Uridine diphosphate glucuronosyl transferase 1A1
WES: Whole-exome sequencing

## References

[1] Perrotta S, Gallagher PG, Mohandas N (2008). Hereditary spherocytosis. Lancet.

[2] Bolton-Maggs PH, Langer JC, Iolascon A, Tittensor P, King MJ, General Haematology Task Force of the British Committee for Standards in Haematology (2012). Guidelines for the diagnosis and management of hereditary spherocytosis--2011 update. Br J Haematol.

[3] Yi Y, Dang X, Li Y, Zhao C, Tang H, Shi X (2018). Genetic diagnosis and pathogenic analysis of an atypical hereditary spherocytosis combined with UGT1A1 partial deficiency: A case report. Mol Med Rep.

[4] Ma S, Deng X, Liao L, Deng Z, Qiu Y, Wei H (2018). Analysis of the causes of the misdiagnosis of hereditary spherocytosis. Oncol Rep.

[5] Andolfo I, Russo R, Gambale A, Iolascon A (2016). New insights on hereditary erythrocyte membrane defects. Haematologica.

[6] He Y, Jia S, Dewan RK, Liao N (2017). Novel mutations in patients with hereditary red blood cell membrane disorders using next-generation sequencing. Gene.

[7] Lin PC, Chiou SS, Lin CY, Wang SC, Huang HY, Chang YS (2018). Whole-exome sequencing for the genetic diagnosis of congenital red blood cell membrane disorders in Taiwan. Clin Chim Acta.

[8] Fan LL, Liu JS, Huang H, Du R, Xiang R (2019). Whole exome sequencing identified a novel mutation (p.Ala1884Pro) of β-spectrin in a Chinese family with hereditary spherocytosis. J Gene Med.

[9] Sriswasdi S, Harper SL, Tang HY, Gallagher PG, Speicher DW (2014). Probing large conformational rearrangements in wild-type and mutant spectrin using structural mass spectrometry. Proc Natl Acad Sci U S A.

[10] Park J, Jeong DC, Yoo J, Jang W, Chae H, Kim J (2016). Mutational characteristics of ANK1 and SPTB genes in hereditary spherocytosis. Clin Genet.

[11] Hassoun H, Vassiliadis JN, Murray J, Yi SJ, Hanspal M (1995). Molecular basis of spectrin deficiency in beta spectrin Durham. A deletion within beta spectrin adjacent to the ankyrin-binding site precludes spectrin attachment to the membrane in hereditary spherocytosis. J Clin Invest.

[12] Agarwal AM, Nussenzveig RH, Reading NS, Patel JL, Sangle N, Salama ME (2016). Clinical utility of next-generation sequencing in the diagnosis of hereditary haemolytic anaemias. Br J Haematol.

[13] Muramatsu H, Okuno Y, Yoshida K, Shiraishi Y, Doisaki S, Narita A (2017). Clinical utility of next-generation sequencing for inherited bone marrow failure syndromes. Genet Med.

[14] Richards Sue, Aziz Nazneen, Bale Sherri, Bick David, Das Soma, Gastier-Foster Julie, Grody Wayne W., Hegde Madhuri, Lyon Elaine, Spector Elaine, Voelkerding Karl, Rehm Heidi L. (2015). Standards and guidelines for the interpretation of sequence variants: a joint consensus recommendation of the American College of Medical Genetics and Genomics and the Association for Molecular Pathology. Genetics in Medicine.

[15] Reithmeier RA, Casey JR, Kalli AC, Sansom MS, Alguel Y, Iwata S (2016). Band 3, the human red cell chloride/bicarbonate anion exchanger (AE1, SLC4A1), in a structural context. Biochim Biophys Acta.

[16] Dhermy D, Galand C, Bournier O, Boulanger L, Cynober T, Schismanoff PO (1997). Heterogenous band 3 deficiency in hereditary spherocytosis related to different band 3 gene defects. Br J Haematol.

[17] Lima PR, Sales TS, Costa FF, Saad ST (1999). Arginine 490 is a hot spot for mutation in the band 3 gene in hereditary spherocytosis. Eur J Haematol.

[18] Russo R, Andolfo I, Manna F, Gambale A, Marra R, Rosato BE (2018). Multi-gene panel testing improves diagnosis and management of patients with hereditary anemias. Am J Hematol.

[19] He BJ, Liao L, Deng ZF, Tao YF, Xu YC, Lin FQ (2018). Molecular Genetic Mechanisms of Hereditary Spherocytosis: Current Perspectives. Acta Haematol.

